# Innovation network, knowledge absorption ability, and technology innovation performance——An empirical analysis of China’s intelligent manufacturing industry

**DOI:** 10.1371/journal.pone.0293429

**Published:** 2023-11-10

**Authors:** Yawei Wang, Yuan Zhou

**Affiliations:** 1 School of Information, Beijing Wuzi University, Beijing, China; 2 School of Public Policy and Management, Tsinghua University, Beijing, China; Nanjing Audit University, CHINA

## Abstract

Based on the social network theory, this study utilizes knowledge absorption capacity as the mediating variable and technology turbulence as the moderating variable; furthermore, it focuses on China’s intelligent manufacturing industry data to explore the effect of the intelligent manufacturing enterprise innovation network on technology innovation performance and the regulating mechanism of technology turbulence. Based on the patent data obtained from Derwent Database (survey period: 2016–2020), the empirical analysis indicates the following: (1) Network relationship, network location, and network density are significantly and positively correlated with technology innovation performance; however, network size exerts no significant effect on technology innovation performance. (2) Network relationship strength, network location, and network density exert significantly positive effects on the two dimensions of knowledge absorption capacity, namely the In-degree and the Out-degree. Network size exerts no significant effect on knowledge absorption capacity. (3) Knowledge absorption capacity exerts a partial mediating effect on the relationship between innovation network and technology innovation performance. (4) The three dimensions of innovation network that exert a significant effect on technology innovation performance are positively correlated with the interaction terms of technology turbulence, which indicates that the interaction terms, namely innovation network and technology turbulence, exert a positive impact on technology innovation performance through knowledge absorption capacity, and that the moderating effect of technology turbulence exerts a role through knowledge absorption capacity. Finally, this study postulates implementations and policy proposals for enhancing the innovation performance of intelligent manufacturing enterprises.

## Introduction

Intelligent manufacturing, the focus of the deep integration of information technology and industrialization, exerts a positive role in promoting China’s development towards an manufacturing power. The "Made in China 2025" proposal has exceedingly promoted the development of intelligent manufacturing in China. Currently, product diversity and knowledge diversity, proliferation, and integration are crucial for realizing intelligent manufacturing [1]. The development of intelligent manufacturing, which is an innovative manufacturing paradigm based on intelligent technology [2] and a key driving force for global industrial innovation [3], has undoubtedly become an inherent demand for the global manufacturing industry [4] furthermore, innovation has become the main driving force for the development of the aforementioned industry [5]. The government has actively issued a series of policies to promote the networked development of innovation. In October 2018, “the Opinions of The State Council on Promoting high-quality development of innovation and Entrepreneurship to create an upgraded version of Mass Entrepreneurship and Innovation” clearly stipulated that enterprises should actively promote the online–offline combination, thus, the leadership in technological innovation can be strengthened, and an integrated innovation mode can be built. Therefore, it is apparent that the intelligent manufacturing enterprises can acquire new knowledge and enhance their technology innovation performance through external cooperation [6, 7]. The report of the 20th National Congress of the Communist Party of China has stipulated significant strategies for promoting the high-end, intelligent, and green development of the manufacturing industry. “Made in China 2025” proposes that enhancing the level of physical and technological innovation, which represents the strategic task, is key to the development of China’s intelligent manufacturing industry. It is apparent that technological innovation can crucially facilitate the development of intelligent manufacturing. Currently, academic research on intelligent manufacturing mainly focuses on the technology empowerment and model construction of intelligent manufacturing in different industries. In the early developmental stage, scholars, who believed that intelligent manufacturing has enhanced enterprise productivity, focused on its impact on enterprise efficiency; however, they have not ascertained whether intelligent manufacturing can promote productivity in all industries [8], as well as the application of industrial robots in the intelligent manufacturing industry [9]. Scholars have analyzed the application of industrial robots in the intelligent manufacturing industry [9] and predicted the future development of intelligent manufacturing, believing that the future manufacturing industry will be composed of a human–robot operators hybrid system [10]. Subsequently, scholars focus on the application of intelligent manufacturing in different fields [11], including the medical industry [12] and emerging technology industries such as 3D printing [13], while analyzing the methods through which enterprises can obtain financial benefits and the upgrading of management models in intelligent transformation [14]. In addition, enhancing intelligent manufacturing technology and user security through mathematics and computer algorithms [15] is also a key consideration for scholars analyzing the mid-term development of intelligent manufacturing. In the past two years, research has focused on the application of industrial big data in intelligent decision-making and the corresponding challenges [16]. Big data driven analysis enhances the market competitiveness of the manufacturing industry by mining the value and potential ability of tacit knowledge therein [17]. The application technology of intelligent manufacturing enhances the remanufacturing accuracy. The balanced decision-making, economic performance, and enthusiasm for participating in remanufacturing under different remanufacturing models have also attracted immense research attention [18]. Intelligent manufacturing technology is also considered as an opportunity for economic recovery and growth after the COVID-19 epidemic [19].

Chinese manufacturers can quickly acquire domain expertise through the application of intelligent manufacturing technology, and the turbulence in the transformation stage may offer a valuable opportunity for leapfrog development. Therefore, exploring the influencing factors pertaining to the development of intelligent manufacturing innovation technology is conducive to addressing development bottlenecks and effecting timely measures [20]. Scholars have gradually discovered the key role of knowledge driven in the process of researching intelligent manufacturing [21]. In practice, enterprises have also realized that by building innovation networks with other organizations, they can continuously explore, absorb, and diffuse new technological knowledge, which ultimately enhances competitiveness [6]. Based on the network theory, the connection strength of network members particularly affects the breakthrough innovation of enterprises [20]. Innovation network-based value creation is a deepening of the value chain theory, which is a fusion of vertical and horizontal development models. Due to the continuous evolution and development of innovation and the value theory, the impact of innovation networks on organizations gradually forms three dimensions: "relationship", "structure", and "location", and innovation networks affect organizational knowledge activities and value creation in different dimensions [22]. The interaction, cooperation, and other practical activities among members of the innovation network directly affect the development, effectiveness, and knowledge transformation of intelligent manufacturing industry technology, and other innovative values [23]. Furthermore, knowledge, which is the most essential competitive capital, is advantageous to enterprises [24]. The ability to absorb knowledge determines the efficiency pertaining to the organizational transformation of novel technologies [25], and the ability to absorb knowledge is the source of organizational innovation [26], which crucially facilitates the innovation performance of enterprises [27]. Currently, some scholars have observed that the knowledge absorption capacity of enterprises may significantly affect the relationship between innovation networks and the technological innovation of enterprises [28]. Scholars have realized the crucial role of knowledge absorption ability in the relationship between innovation networks and technological innovation performance, and they have also conducted corresponding research. Some scholars have explored the mediating role of knowledge absorption ability in the impact of network density on enterprise innovation performance [29]. Moreover, some studies analyze the role that knowledge absorption ability exerts on the relationship between network characteristics (i.e., network size and homogeneity) and innovation performance [30]. Network density determines the depth and breadth of knowledge exchange among network members, thereby affecting the innovation performance of knowledge receivers [31]. In the past two years, studies have begun to explore the intermediate effects of knowledge absorption capacity under the innovation model of industries, universities, and research [32]. The centrality and density of the overall network level positively affect innovation performance [33]. As the network scale expands, the structure of the innovation service network exhibits a small-world characteristic; furthermore, the absorption capacity and openness of the innovation subjects in the innovation network change with the change of knowledge stock [34], and the network model directly determines the overall knowledge stock of the network.

Based on the preceding literature analysis, scholars mainly analyze innovation characteristics from three dimensions, namely connection strength, network density, and network size. In addition, the social network theory and the knowledge-based theory indicate that a crucial feature of innovation networks is knowledge flow (i.e., knowledge sharing, acquisition, and utilization within the network) [35]. Therefore, knowledge should crucially facilitate the process of influencing enterprise innovation performance through the characteristics of innovation networks [31]. Apparently, current research mainly explores the intermediate role of knowledge absorption capacity of the impact of a certain dimension of the innovation network on innovation performance, without conducting a systematic analysis from the overall perspective.

Based on the previous analysis, innovation networks and knowledge absorption capabilities crucially affect the technology development of the intelligent manufacturing industry; however, there is a lack of research in this field. In addition, technological turbulence refers to the uncertainty, complexity, and unpredictability of technological changes. The turbulent technological environment introduces a certain degree of difficulty, which limits enterprise innovation and presents new challenges [36]; moreover, external technological uncertainty exerts a moderating effect on the impact of technological learning and management learning on breakthrough innovation [37]. Meanwhile, patents are being increasingly utilized to measure the degree of organizational innovation. Therefore, this study represents innovation networks with patent citation networks, analyzes the impact of innovation networks on the development of technological innovation in the intelligent manufacturing industry, studies the knowledge interaction between organizations in innovation networks, explores the mediating role of knowledge absorption capacity and the moderating effect of technology turbulence, and proposes policy recommendations for industrial development. This study analyzes the internal impact mechanism of innovation development of China’s intelligent manufacturing industry, helps intelligent manufacturing enterprises diagnose problems in innovation and development in time, promotes enterprises to position innovation strategies with systematic thinking, and puts forward targeted suggestions for enterprises.

## Literature review and research assumptions

### Innovation network and technology innovation performance

The innovation network was first defined by Freeman as a basic institutional system for addressing systemic innovation, which is primarily connected through innovation cooperation between organizations [38]. Subsequently, Ferrary further expanded the meaning of innovation network to create novel business processes or products, ultimately revolutionizing the original value chain as a business network [39]. The value of innovation networks lies in stimulating technological innovation in the industry, emphasizing the complementarity, competition, and cooperation of various members in the network, while possessing the special attributes and resource heterogeneity of complex networks [40]. The research results of the past two years also indicate that innovation networks not only stem from business goals, but from social goals, and can still create business opportunities for companies [41]. Some scholars have begun to analyze the impact of node turnover on network evolution based on the dynamics of innovation networks, especially the impact of network evolution on innovation results [42]. Therefore, this article attempts to study its impact on technological innovation performance from the perspective of innovation network development. Based on literature analysis, this article divides innovation networks into three dimensions: network relationship [6] relationship structure [43], and network location [44], and explores the relationship between each dimension and technological innovation performance.

Network relationship refers to the process through which an organization forms partnerships with members of the network to obtain complementary resources (i.e., novel knowledge and technological innovation) from the outside world. Rojas et al. [45] divided scientific network relationship into two complementary dimensions, namely internal and external, and analyzed the impact of different dimensions on technology innovation performance. Network relationship refer to the most critical network attribute in innovation networks [46], and scholars mostly analyze network relationships based on the relationship strength and relationship quality proposed by Granovetter [46]. It is generally believed that the heterogeneity and stability of network relationships exert a positive promoting effect on enterprises’ acquisition and integration of novel knowledge, and promote exploratory innovation practices [22]. The heterogeneity of network relationships contributes to radical innovation, and organizations can acquire more innovative knowledge by utilizing and maintaining network relationships with network members. Establishing partnerships with heterogeneous, allied members can expand innovation boundaries [47]. In recent years, patents have been utilized as an effective basis for evaluating technological innovation, and patent analysis methods are often utilized to analyze knowledge flow [48]. By analyzing patent and paper data, Pei YL [49] noted that scientific and technological network cooperation exerts a positive effect on organizational technology innovation performance, and they concluded that the organization’s knowledge integration ability exerts a crucial intermediary effect between the industry–university research network relationship and organizational innovation. Therefore, based on the diversity of network knowledge, innovation networks (i.e., an organizational form that integrates implicit and explicit knowledge) are occasioned by a single organization in regard to knowledge creation and integration. An efficient network relationship can promote knowledge sharing among network members, thereby enabling organizations to obtain more new resources, ultimately optimizing existing processes, and developing new products. Based on the existing literature, this article postulates the following hypothesis:

Hypothesis 1: The strength of the network relationship positively affects the technology innovation performance of enterprises.

Network location is the ultimate result of establishing relationships between an organization and other members of the innovation network, reflecting the position of the enterprise in the network and the ability of the organization to absorb and diffuse new knowledge based on its embedded innovation network. The resources and technological knowledge obtained by the organization in the network are its internal driving force through which its own technology innovation performance can be enhanced [44]. Based on different perspectives, the influence of network location on enterprise innovation has been extensively studied. Wan et al. [50] observed that network location reflects resource competition or technological cooperation among network members. Tsai [51] also noted that member organizations with dominant network locations exhibit a stronger ability to acquire and allocate resources compared to other member organizations. Based on empirical analysis pertaining to emerging industries, Lv YB et al. [52] observed that the centrality of network location negatively regulates the effect of inward open innovation on innovation performance, whereas the connectivity of network location exerts a positively regulates the effect of inward open innovation on innovation performance. The social network theory states that using behavioral practices, organizational members in innovation networks acquire complementary innovation resources, and that they utilize their status in the network [47]. The individual differences of the organization determine different resource acquisition capabilities. marginal positions [53], member organizations closer to the network center possess higher industry authority and are the leading enterprises in the industry. Meanwhile, member organizations possess a stronger ability to acquire external knowledge; thus, novel and diversified resource information can be explore in a timelier manner. Their ability to absorb new knowledge is also higher than that of organizations in marginal positions [53]. Network centrality is a key index to evaluate the position of an organization in the innovation network [44]. When the centrality of the network is higher, the central role of the organization in the innovation network is stronger; thus, the organization possesses more opportunities to acquire novel innovative information resources in the network and utilize its own leadership position. Organizations that exhibit a high degree of intermediate centrality can constantly complement resources with other organizations [44], optimize, integrate and apply acquired knowledge, and overcome existing technologies through heterogeneous and diversified knowledge; thus, more technological innovation outputs can be obtained [52]. Therefore, Hypothesis H2 was proposed:

Hypothesis 2: Network centrality exerts a positive influence on the technology innovation performance of enterprises.

Network structure has been defined from different perspectives [54]. Based on the social capital theory, other network members can provide innovative knowledge for organizations, whereas network size and network density exert a positive effect on enhancing the technology innovation performance of organizations [55]. Structural holes and network centrality exert positive effects on the innovation performance of network members; however, the intensity of network density exerts negative effects on the technology innovation performance. Tian et al. [54], who divided the innovation network structure from two dimensions (i.e., openness and inclusiveness), analyzed the relationship between the innovation network of high-tech zones and organizational innovation performance; consequently, they observed that openness exerted a positive promoting effect on organizational innovation performance, whereas the relationship between inclusiveness and organizational innovation performance exhibited an inverted "U" shaped curve.

Numerous scholars have analyzed the influence of network structure on organizational technology innovation performance from two variables, namely resources and capabilities [56]. Based on the resource-based view, the expansion of network scale represents the increase of network resources, while from the perspective of capability, the network density, which represents the degree of close connection between network nodes, can characterize the strength of network innovation. Based on literature review, this study measures network structure from two dimensions: network size and network density [55]. From the resources perspective, organizations can explore diversified resources through a heterogeneous network structure [57]. From the capabilities perspective, organizations can enhance technological innovation, the development of novel products, and obtain more market opportunities based on the network structure [58]. Based on the literature summary, this study measures the network structure from two dimensions, namely network size and network density, and proposes the following hypotheses:

Hypothesis 3a: The network scale positively affects the technology innovation performance of enterprises.
Hypothesis 3b: Network density positively affects the technology innovation performance of enterprises.

### Innovation network and knowledge absorption ability

In regard to the open environment of innovation networks, organizations obtain heterogeneous resources through establishing complementary cooperation with network members, and the acquisition of heterogeneous knowledge is closely related to the organization’s knowledge absorption capacity [59]. Knowledge absorption capacity refers to an organization’s ability to identify, acquire, and absorb external new knowledge and transform it into novel products or services, which is the core factor that indicates the core competitiveness of an organization [60]. Innovation network refers to the construction of a complementary knowledge chain between organizations and external members, and exhibits the following attributes: knowledge flow, transfer, and diffusion [54]. Scholars have analyzed the relationship between innovation network and knowledge absorptive capacity from different perspectives. According to [54], the influence mechanism of innovation network openness and knowledge absorption capacity on organizational innovation performance is verified by analyzing the survey data of 338 enterprises, which indicates that innovation network openness exerts a significantly positive influence on knowledge absorption capacity. Sun et al. [61], who applied the quality perspective, concluded that organizations close to the network center exhibit broader business strategic vision, operational capabilities, and competitive advantages, and can integrate knowledge more effectively.

Specifically, the above research results indicate that strong network relationships can enable organizations to acquire more valuable new knowledge and resources [56], and the enhancement of network scale and density can also promote the diffusion and absorption of knowledge flow in the innovation network [59]. Based on the preceding analysis, hypotheses 4, 5, and 6 are presented:

Hypothesis 4: The network relationship strength positively affects knowledge absorption ability.
Hypothesis 5: The network location centrality positively affects knowledge absorption ability.
Hypothesis 6a: Network size positively affects knowledge absorption capacity.
Hypothesis 6b: Network density positively affects knowledge absorption ability.

### The mediating role of knowledge absorption ability

It is generally believed that the identification, acquisition, and application of external knowledge are positively correlated with organizational performance [62], and the core factor for enhancing organizational technology innovation performance lies in the ability of an organization to acquire scarce knowledge resources in the innovation network [63]. According to [62], the open environment of an innovation network can enable organizations to efficiently acquire complementary innovation resources at a low cost; however, the degree of transformation is dependent on the knowledge absorption capacity. Only by transforming the acquired innovation resources into new products or processes through the knowledge absorption capacity can innovative resources enhance the technology innovation performance of organizations. Therefore, the deep transformation of external new knowledge resources is fundamental to organizational innovation, and this process is closely related to organizational knowledge absorption capacity. Based on the preceding analysis, this study proposes the following hypothesis:

Hypothesis 7: Knowledge absorption ability exerts a mediating role between innovation networks and technology innovation performance.

### The moderating effect of technology turbulence

Technology turbulence refers to the scenario in which industrial technologies exhibit uncertain changes [64], which will impact the current operation and innovation practice of an organization [65]. Cassiman [66] noted that in turbulent environments, organizations usually make more efforts to seek outside knowledge. Thornhill [67] stated that turbulent environments may lead to more productive organizations. In addition, based on the dynamic capability theory, the corresponding products of the organization will be eliminated quickly in the turbulent external environment, which enables the organization to strengthen exploratory learning as a means of acquiring new knowledge away from the existing solidified technology [68]. Garud and Droge [69, 70] believe that the existence of a turbulent technological environment positively affects knowledge transformation, and that this positive effect will be weakened in a stable environment; in a stable environment, the organization’s affinity for external knowledge and the efficiency of generating new knowledge will be reduced. Therefore, this study proposes the following hypothesis:

H8a: Technology turbulence positively moderates the influence of network relations on technology innovation performance through knowledge absorption capacity.
H8b: Technology turbulence positively moderates the influence of network location on technology innovation performance through the knowledge absorption capacity.
H8c: Technology turbulence positively moderates the influence of network size on technology innovation performance through the knowledge absorption capacity.
H8d: Technology turbulence positively moderates the influence of network density on technology innovation performance through the knowledge absorption capacity.

## Research design

### Data and sample selection

Scholars indicate that patents are the characterization of technological innovation and the most powerful tool through which technological innovation can be measured. Numerous studies have analyzed knowledge activities among enterprises and organizations based on patent analysis [59]. Therefore, this study builds an innovation network based on patent data. The Derwent Innovations Index (DII) patent database (patent matching accuracy: >99.9%) is integrated with the Derwent World Patent Index (DWPI, Derwent Patents Index, and Derwent Patents Citation Index (PCI), a database consisting of more than 18 million essential invention patents. For more than 38.9 million patent information, covering 41 different patent offices worldwide (i.e., more than 100 countries, including China’s utility Model patent information), patent data can be traced back to 1963. In regard to the periodicity of DII patent data collection with a lag period of approximately 18 months, this study collected global intelligent manufacturing patent data from 2010 to 2020 using intelligent manufacturing keywords, and utilized the period from 2016 to 2020 as the benchmark data for the construction of an innovation network. Search keywords for related patents are derived from academic literature, expert consultations, policy documents, and industry reports, and they include the following: intelligent manufacturing; smart manufacturing; intelligence manufacturing; intelligence manufacturing; intellectual manufacturing; intellectual manufacturing; smart manufacture; intelligent manufacturing; intelligent development; intelligent product; and smart manufacturing. A total of 19,976 pieces of Chinese intelligent manufacturing patent data were collected herein. Moreover, through patent data cleaning and patent number ranking, 80 Chinese intelligent manufacturing sample enterprises were selected to build an intelligent manufacturing innovation network (patent citation network). Multiple linear regression analysis and empirical verification will be conducted through the panel data of the 80 intelligent manufacturing enterprises from 2016 to 2020. The selection principles are as follows: (1) The number of patents ranked from the top to the bottom; (2) representative enterprises within the industry; (3) spanning different fields of intelligent manufacturing technology; and (4) industry report analysis and expert recommendation.

### Variable and measurement

This study utilizes Thomson Data Analyzer (TDA) social network analysis software to plot the patent cooperation network for each year, and, subsequently, utilizes UCINET software to calculate the network relationship strength, intermediate centrality, network size and network density, and node out-degree and in-degree of these patent reference networks; thus, the measurement indicators of these variables is obtained.

Herein, the patent cooperation network was plotted by the Thomson Data Analyzer (TDA) social network analysis software, and the UCINET software was utilized to calculate the following measurement indicators: network relationship strength, intermediate centrality, network size and network density, and node out-degree and in-degree.

(1) Dependent variables
  Based on the literature review, this study selects enterprise technology innovation performance (PF) as the dependent variable. Because patent applications may fail, the utilization of application data alone cannot characterize the technology transformation output of a company. However, the Derwent Innovations Index (DII) contains approved patent data. Therefore, this study selects the patent data included in the DII of the company in year t to measure the company’s technology innovation performance.
(2) Independent variables

Based on the preceding literature analysis, this study divides innovation network into four dimensions, namely network relation (RS), network location (BC), network size (SC), and network density (DE), and utilizes them as independent variables; thus, their effects on dependent variables are respectively analyzed. The specific measurements of independent variables are as follows:

Although questionnaires are widely utilized by contemporary scholars as the main tool for measuring network relations, knowledge flow in the innovation network is bidirectional, and the object of the questionnaire, which often fails to investigate partners simultaneously, is only sample enterprises [22]. In addition, the questionnaire method is highly subjective. In the innovation network, the higher the frequency of patent citations between two nodes, the stronger the cooperation, communication, and correlation between the two nodes. Therefore, the strength of the innovation network (RS) relationship refers to the average strength of all direct connections in an organization’s network, namely the average frequency of direct connections with the enterprise [7].

Intermediate centrality is utilized to measure the strength of the network subject’s control over network resources, and represents the shortest path frequency of any pair of subjects connected through the subject [48]. In the network, the intermediate centrality of the member body reflects the degree of control of network resources by the node enterprise, which is utilized to measure the network location (BC) herein.

In the process of building innovation networks, isolated points often exist; enterprises that are not connected with any nodes should be proposed. Therefore, the network size (SC) refers to the number of node enterprises that can be connected with other members [48].

Network density (DE) refers to the ratio of the actual connection frequency between network nodes to the number of possible connections in the network; thus, it represents the strength of the knowledge flow in the innovation network [59].

(3) Intermediate variables
  The knowledge absorption capacity of an enterprise is defined as the ability of an organization to acquire, accept, transform, and output knowledge. The knowledge absorption process includes not only self-transformation but also knowledge spillover. Therefore, in-degree (ID) is utilized to measure the ability of knowledge transformation, and out-of-degree (OD) is utilized to measure the ability of an enterprise to export knowledge. In the patent citation networks, the line points to the node, which indicates that the node introduces the patent. Conversely, the lines sent by the node represents the patent output. Therefore, the in-degree can be obtained by calculating the frequency of the lines pointing to the node organization in the innovation network (i.e., patent citation network). The out-degree is obtained by calculating the number of connections to other members organized by nodes [48, 59].
(4) Moderator variables
  The knowledge absorption capacity of an organization is often affected by the uncertainty of the technological environment. Therefore, according to [6], technology turbulence is utilized to measure the uncertainty of technological environment using materials. Herein, the number of intelligent manufacturing industries is utilized as the dependent variable and the year as the independent variable for regression, and the obtained regression coefficient is standardized. Subsequently, the standard deviation is divided by the average number of patents in the whole industry, and the final standardized coefficient is utilized to measure the technology turbulence. The greater the value, the stronger the uncertainty pertaining to the technical environment of the intelligent manufacturing industry [6].
(5) Control variables
  This study introduces enterprise age, R&D investment, nature of property rights [71] and patent inventory [6] as control variables, and enterprise age (AG) refers to the time period from the establishment of the enterprise to year t. Patent Inventory (PS) refers to the number of patents related to intelligent manufacturing in a company before year t (tracing back to the initial year “2010” when the sample data was collected); thus, the existing technology innovation performance of the organization is controlled. R&D investment(RD) refers to the logarithm of the sum of R&D amount and 1 [71]. The nature of property rights (NP)is measured by virtual variables. If the enterprise is a state-owned enterprise, the value is 1, and if it is a non-state-owned enterprise, the value is 0 [35].

### Regression models

To examine the impact of the dimensions of innovation networks on technology innovation performance, the study utilized a panel model that refers to the study conducted by Liang Y et al. [6], Qian X [59] and Ping L et al. [63].

(1) Construction of a mediation model for knowledge absorption capacity.

First, to examine the impact of enterprise innovation networks on enterprise technology innovation performance (PF), hypotheses 1, 2, 3a, and 3b are proposed; furthermore, the models constructed herein are as follows:

$${PF}_{i,t}=e_{0}+e_{1}{RS}_{i,t}+e_{2}{BC}_{i,t}+e_{3}{SC}_{i,t}+e_{4}{DE}_{i,t}+\varepsilon$$ (Model 5)

Second, to test the impact of various dimensions of enterprise innovation network on knowledge absorption capacity, the following two models are constructed based on hypotheses 4, 5, 6a, and 6b.


$${ID}_{i,t}=a_{0}+a_{1}{RS}_{i,t}+a_{2}{BC}_{i,t}+a_{3}{SC}_{i,t}+a_{4}{DE}_{i,t}+\varepsilon$$ (Model 1)

$${OD}_{i,t}=b_{0}+b_{1}{RS}_{i,t}+b_{2}{BC}_{i,t}+b_{3}{SC}_{i,t}+b_{4}{DE}_{i,t}+\varepsilon$$ (Model 2)

The third step entails testing the mediating effect of knowledge absorption capacity. Based on Hypothesis 7, the following three models are designed.


$${PF}_{i,t}=f_{0}+f_{1}{RS}_{i,t}+f_{2}{BC}_{i,t}+f_{3}{SC}_{i,t}+f_{4}{DE}_{i,t}+f_{5}ID{ID}_{i,t}+\varepsilon$$ (Model 6)

$${PF}_{i,t}=g+g_{1}{RS}_{i,t}+g_{2}{BC}_{i,t}+g_{3}{SC}_{i,t}+g_{4}{DE}_{i,t}+g_{5}{OD}_{i,t}+\varepsilon$$ (Model 7)

$${PF}_{i,t}=h_{0}+h_{1}{RS}_{i,t}+h_{2}{BC}_{i,t}+h_{3}{SC}_{i,t}+h_{4}{DE}_{i,t}+h_{5}{ID}_{i,t}+h_{6}{OD}_{i,t}+\varepsilon$$ (Model 8)

In addition, for comparative analysis, this study tests the influence of knowledge absorption capacity on technology innovation performance (PF). Based on Hypothesis7, this study constructs the following two models.


$${PF}_{i,t}=c_{0}+c_{1}{ID}_{i,t}+\varepsilon$$ (Model 3)

$${PF}_{i,t}=d_{0}+d_{1}{ID}_{i,t}+\varepsilon$$ (Model 4)

(2) The moderating effect of technology turbulence

To test the moderating effect of technology turbulence on the relationship between innovation networks and enterprise technology innovation performance (PF) through the knowledge absorption capacity (i.e., to test the influence of moderating variables on the relationship between independent and dependent variables), this study refers to the testing program designed by Su and Li [72]; thus, Hypothesis 8a to Hypothesis 8d are verified. The models are as expressed as follows:

$$PF=n_{0}+n_{1}RS+n_{2}BC+n_{3}SC+n_{4}DE+n_{5}UT+n_{6}{RSxUT}_{i,t}+n_{7}{BCxUT}_{i,t}+n_{8}{SCxUT}_{i,t}+n_{9}{DExUT}_{i,t}+\varepsilon$$ (Model 11)

$${ID}_{i,t}=l_{0}+l_{1}{RS}_{i,t}+l_{2}{BC}_{i,t}+l_{3}{SC}_{i,t}+l_{4}{DE}_{i,t}+l_{5}{UT}_{i,t}+l_{6}{RSxUT}_{i,t}+l_{7}{BCxUT}_{i,t}+l_{8}{SCxUT}_{i,t}+l_{9}{DExUT}_{i,t}+\varepsilon$$ (Model 9)

$${OD}_{i,t}=m_{0}+m_{1}{RS}_{i,t}+m_{2}{BC}_{i,t}+m_{3}{SC}_{i,t}+m_{4}{DE}_{i,t}+m_{5}{UT}_{i,t}+m_{6}{RSxUT}_{i,t}+m_{7}{BCxUT}_{i,t}+m_{8}{SCxUT}_{i,t}+m_{9}{DExUT}_{i,t}+\varepsilon$$ (Model 10)

$${PF}_{i,t}=q_{0}+q_{1}{RS}_{i,t}+q_{2}{BC}_{i,t}+q_{3}{SC}_{i,t}+q_{4}{DE}_{i,t}+q_{5}{ID}_{i,t}+q_{6}{OD}_{i,t}+q_{7}{UT}_{i,t}+q_{8}{RSxUT}_{i,t}+q_{9}{BCxUT}_{i,t}+q_{10}{SCxUT}_{i,t}+q_{11}{DExUT}_{i,t}+q_{12}{IDxUT}_{i,t}+q_{13}{ODxUT}_{i,t}+\varepsilon$$ (Model 12)

The variables utilized in the preceding model and their respective calculation methods have been explained in a preceding section; this notwithstanding, i denotes the sample individual, and t denotes the year.

## Empirical results

### Descriptive statistics

First, the results pertaining to the descriptive statistical analysis of variables are depicted in Table 1, in which the mean value, variance inflation factor (VIF), standard deviation, and correlation coefficient between variables are utilized to detect whether collinearity between variables exists. Both sample quantity and sampling error can affect the significance of correlation; therefore, there is causal relationship between variables, and not necessarily significant correlation. Meanwhile, if the correlation coefficient between variables is greater than 0.75, there may be collinearity between the two variables [58]. In Table 1, the maximum value of VIF is 2.037 (a <5 coefficient often indicates no collinearity [6]). Table 1 reveals that the correlation coefficients of all variables are less than 0.75, which indicates that there is no collinearity problem for each variable in this study. In addition, Table 1 indicates that the independent variables (i.e., network relationship strength, network location, network density, and network size) exhibit a significantly positive correlation with technology innovation performance, in-degree and out-degree respectively, whereas in-degree and out-degree exhibit a significantly positive correlation with technology innovation performance, respectively. Apparently, the results of the descriptive statistical analysis correspond to the conclusions of the preceding theoretical derivation and are consistent with the general objective facts.

**Table 1:** Descriptive statistics and correlation coefficients of variables.

Variables	Mean	Std. -Dev	VIF	PF	RS	BC	SC	DE	ID	OD	UT	AG	PS	NP	RD
PF	7.901	8.963		1											
RS	12.845	14.612	1.102	0.309*	1										
BC	9.099	12.251	1.163	0.498*	0.103	1									
SC	70.815	1.119	2.135	0.564*	0.162	0.801	1								
DE	0.612	1.037	1.742	0.630*	0.087	0.032`	0.201	1							
ID	31.784	8.421	1.191	0.572*	0.408*	0.418*	0.398*	0.349*	1						
OD	28.763	9.957	2.562	0.493*	0.557*	0.491	0.412*	0.509*	0.211*	1					
UT	0.209	0.312	1.346	0.219	0.451	0.503	0.612	0.478	0.501	0.178	1				
AG	24.191	13.813	1.298	0.101	0.187	0.057	0.070	0.082	0.092	0.152	0.004	1			
PS	56.384	7.098	1.402	0.234	0.309	0.211	0.195	0.143	0.426	0.213	0.094	0.104	1		
NP	0.376	0.432	0.301	0.204	0.108	0.217	0.092	0.068	0.089	0.095	0.117	0.101	0.210	1	
RD	19.026	15.602	1.219	0.192*	1.067	2.234	1.920	1.171	0.902*	0.123	1.324	1.092	0.131	0.113	1

### Regression analysis

Herein, mixed effects and fixed effects are selected using a likelihood ratio test (L.R test). If fixed effects are determined, random effects and fixed effects are selected by the Hausman test. The test results are illustrated at the bottom of Table 2. Table 2 indicates that the L.R test of all models is below the 1% significance level, which indicates that these models have passed the test and the mixed effect model has been eliminated [6]. Therefore, the fixed effect model is selected for all models.

**Table 2:** Regression analysis pertaining to the fixed effect model of panel data.

	Model1	Model 2	Model 3	Model 4	Model 5	Model 6	Model 7	Model 8
Variables	ID	OD	PF	PF	PF	PF	PF	PF
Independent variables								
RS	0.407** (0.019)	0.423** (0.021)			0.398* (0.034)	0.361* (0.025)	0.385* (0.037)	0.303** (0.009)
BC	0.375* (0.026)	0.319** (0.017)			0.372* (0.052)	0.463** (0.029)	0.371* (0.034)	0.289** (0.019)
SC	0.231 (0.008)	0.313 (0.012)			0.241 (0.013)	0.198 (0.024)	0.107 (0.021)	0.009 (0.092)
DE	0.208** (0.015)	0.297* (0.019)			0.321** (0.009)	0.256** (0.014)	0.309* (0.024)	0.211* (0.018)
Mediating variable								
ID			0.319** (0.054)			0.324*** (0.071)		0.367** (0.046)
OD				0.401** (0.012)			0.432** (0.071)	0.310* (0.029)
Control variable								
AG	0.017 (0.009)	0.087 (0.008)	0.211 (0.013)	0.127 (0.024)	0.099 (0.002)	0.085 (0.028)	0.090 (0.013)	0.074 (0.011)
PS	0.041 (0.003)	0.126 (0.016)	0.089 (0.007)	0.105 (0.017)	0.198 (0.014)	0.190 (0.056)	0.261 (0.017)	0.186 (0.019)
NP	0.038 (0.031)	0.091 (0.039)	0.037 (0.013)	0.104 (0.019)	0.112 (0.033)	0.091 (0.009)	0.137 (0.020)	0.063 (0.006)
RD	0.309* (0.015)	0.298 (0.013)	0.431* (0.015)	0.487** (0.017)	0.398* (0.021)	0.491** (0.016)	0.503* (0.026)	0.307** (0.017)
R^2^	0.398	0.437	0.422	0.398	0.499	0.574	0.587	0.693
DW	2.186	2.109	2.047	2.112	2.098	2.125	1.203	1.906
LR Test	1.354***	1.798***	1.865***	1.799***	2.099***	2.112***	1.897***	2.001***
Hausman Test	3.089***	5.907***	7.531***	7.901***	6.386***	8.990***	13.812***	17.451***
Model	Fixed	Fixed	Fixed	Fixed	Fixed	Fixed	Fixed	Fixed

Note

* p≤ 0.05 (double tailed test)

** P≤ 0.01 (double tailed test); and

*** P ≤ 0.001 (double tailed test)

### Verification of the relationship between innovation network, knowledge absorption capacity, and technology innovation performance

Herein, multiple linear regression models 1 to 8 are constructed; thus, the aforementioned theoretical assumptions are verified (Table 2). Model 5 indicates that network relationship strength (RS), network location (BC), and network density (DE) exert significant effects on technology innovation performance (p≤0.05), whereas network size (SC) exerts no significant impact on technology innovation performance (PF). Therefore, the empirical results support hypotheses 1, 2, and 3b, but not Hypothesis 3a. The results indicate that strengthening the interaction intensity between organizations and network members, strengthening the centrality of organizations in the network, and strengthening the intensity of network knowledge flow are conducive to enhancing the technology innovation performance (PF) of organizations. However, the number of nodes in the network (network size) exerts no positive impact on technology innovation performance (PF), which may be rationalized as follows: due to the intensification of knowledge flow, the number of enterprises that exhibit isolated points is gradually decreasing. Therefore, the network size is basically stable and will not exert a significant impact on the innovation network.

Models 1 and 2 indicate that network relationship strength (RS), network location (BC, and network density (DE) exert significantly positive effects on the two dimensions of knowledge absorption capacity. Therefore, the empirical results support Hypothesis4, Hypothesis 5, and Hypothesis 6b. Network size (SC) exerts no significant influence on knowledge absorption capacity; therefore, Hypothesis H6a fails the test. This conclusion verifies the aforementioned theoretical derivation, and the knowledge flow of innovation network exerts a positive role in promoting technological innovation [73].

Models 3 and 4 indicate that the two dimensions of knowledge absorption capacity, namely the In-degree (ID) and the Out-degree (OD), respectively, exert a significantly positive impact on technology innovation performance (PF). Models 6 and 7 indicate that knowledge absorption ability exerts a significant mediating effect on the impact of the innovation network (RS, BC, and DE) on technology innovation performance (PF); thus, the results support hypothesis 7. The values of R^2^ in Model 6 and Model 7 are greater than that in models 1 and 2, which also verifies the rationality of the mediating effect. The network size (SC) does not exert a significant impact on knowledge complementarity and technology innovation performance (PF) due to the frequent interaction and increased awareness of cooperation among participants, as well as the reduction of isolated enterprises.

Model 8 comprehensively demonstrates the relationship between innovation network, knowledge absorption capacity, and technology innovation performance. The results indicate that after the introduction of the two dimensions of knowledge absorption capacity, the correlation coefficient of the innovation network’s influence on technology innovation performance decreases significantly, which further verifies the intermediary effect. It can be observed that the network relationship (RS) exerts the greatest influence on technology innovation performance (correlation coefficient is 0.303**), and the network density (DE) exerts the least influence (correlation coefficient is 0.211*), whereas the network size (SC) exerts no significant influence on technology innovation performance (PF).

### The moderating effect of technology turbulence (UT)

To verify hypothesis H8, the technology turbulence (UT) of the regulating variable should be tested, and, subsequently, the knowledge absorption capacity of the intermediate variable should also be tested. The results are depicted in Table 3. According to the testing process of the moderating effect with mediating variables, this study first tests whether the cross-multiplication coefficient between innovation network and technology turbulence depicted in Model 11 is significant. If it is significant, the next test will be conducted; otherwise, the test will be stopped. As illustrated in Model 11, except for network size (SC), the coefficients of cross-pollination terms between innovation network and technology turbulence (UT) are all positive, and pass the test at the 10% significant level, which indicates that technology turbulence positively moderates the influence of the innovation network on technology innovation performance (PF); additionally, this result indicates that the turbulent technological environment can promote the interaction among the members of intelligent manufacturing enterprises and expand the network’s innovation effect. The subsequent comparative analysis indicates that the coefficient of the cross term between the innovation network and technology turbulence is significantly positive in models 9 and 10. And the regression coefficient of knowledge absorption capacity and technology innovation performance is also significantly positive in Model 12; thus, the intermediate variable “knowledge absorption capacity” is added. Therefore, it can be inferred that the moderating model with intermediate variable is valid. However, in Model 12, the regression coefficient of the cross-multiplication term between innovation network and technology turbulence is not significant. Therefore, it can be inferred that the moderating effect of technology turbulence is realized through the intermediary variable of knowledge absorption capacity. The empirical results support Hypothesis 8, which indicates that the innovation of enterprises is dependent on the integration of internal and external knowledge and technology. When the technological environment of the industry is in a state of high speed and turbulence, the rapid technological progress significantly affects the life cycle of existing products, increases the rate at which the currently advantageous products or services become obsolete, weakens the differentiation advantage that has been formed, and constantly overcomes the competitive barriers. In this environment, by integrating internal and external technologies, enterprises can continuously introduce novel products, expand product or service fields, and translate innovation network effects into technology innovation performance with more ease. In addition, it has been verified that network size (SC) exerts no significant influence on innovation network and technology innovation performance (PF), which indicates that Hypothesis 8c is not valid.

**Table 3:** The moderating effect of technology turbulence.

	Model 9	Model 10	Model 11	Model 12
Variables	ID	OD	PF	PF
RS	0.503* (0.023)	0.541** (0.013)	0.409** (0.032)	0.587*** (0.008)
BC	0.405** (0.011)	0.413** (0.023)	0.309* (0.028)	0.472** (0.017)
SC	0.432 (0.013)	0.482 (0.017)	0.351 (0.009)	0.506 (0.011)
DE	0.301** (0.017)	0.265** (0.022)	0.299* (0.011)	0.398** (0.023)
ID				0.387** (0.018)
OD				0.409* (0.017)
IDxUT				0.311 (0.010)
ODxUT				0.343 (0.021)
UT	0.281* (0.021)	0.321* (0.010)	0.387* (0.026)	0.390** (0.032)
RSxUT	0.209* (0.012)	0.197* (0.014)	0.242* (0.019)	0.212 (0.026)
BCxUT	0.199* (0.015)	0.214* (0.026)	0.238* (0.014)	0.317 (0.029)
SCxUT	0.211 (0.009)	0.192 (0.014)	0.225 (0.017)	0.265 (0.025)
DExUT	0.243** (0.023)	0.267* (0.016)	0.347* (0.014)	0.379 (0.027)
Control variable				
AG	0.163 (0.021)	0.190 (0.014)	0.145 (0.009)	0.209 (0.013)
PS	0.207 (0.009)	0.167 (0.004)	0.090 (0.008)	0.104 (0.011)
NP	0.113 (0.009)	0.283 (0.012)	0.171 (0.011)	0.293 (0.008)
RD	0.201* (0.019)	0.342 (0.013)	0.329 (0.011)	0.296 (0.027)
R^2^	0.428	0.519	0.547	0.663
DW	2.117	2.098	1.838	1.901
LR Test	1.935***	1.864***	1.799***	1.869***
Hausman Test	6.064***	7.653***	7.423***	8.367***
Model	Fixed	Fixed	Fixed	Fixed

Note

* p≤ 0.05(double tailed test)

** P≤ 0.01 (double tailed test); and

*** P ≤ 0.001 (double tailed test)

When conducting a statistical regression model, this study adopted a gradual addition of variables. Thus, it compared and analyzed the impact of independent variables on technological innovation performance; the impact of independent variables and intermediary variables on technological innovation performance; and the impact of independent variables, intermediary variables, and moderating variables on technological innovation performance, simultaneously. Therefore, the impact of variable introduction on empirical analysis results can be effectively analyzed. Comparing Tables 2 and 3, the change trend of R^2^ demonstrates the rationality of the final model (Model 12).

## Conclusion and policy implications

If we consider the intelligent manufacturing industry, this study explores the impact pertaining to various dimensions (i.e., network relationship, network locations, and network structures) of innovation networks (patent citation networks) on technology innovation performance (PF). Based on the social network and knowledge creation theories, the study reveals the impact exerted by the various dimensions of innovation networks on knowledge absorption capacity. Based on the preceding analysis, this study utilizes knowledge absorption capacity as the intermediate path; thus, it analyzes the impact mechanism of innovation networks on the technology innovation performance of intelligent manufacturing enterprises. The main conclusions of this study are as follows:

(1) With the exception of the network size, the dimensions associated with the innovation network of intelligent manufacturing enterprises exert a significantly positive impact on technology innovation performance. With regard to the influence on technology innovation performance, the dimensions are ranked as network relationship strength, network location, and network density, in decreasing order; moreover, network size exerts no significant influence on innovation performance. The network relationship in the innovation network of the intelligent manufacturing industry is reflected as the process pertaining to the complementary learning of node enterprises; the network location is reflected as the process through which node enterprises utilize more opportunities to acquire new knowledge; and the network density is reflected as the intensity of knowledge flow in the innovation network. Specifically, node enterprises in the innovation network acquire, integrate, transform, and create new knowledge through complementary cooperation and learning, and deepen and utilize the new knowledge within the organization [73, 74]. In this process, node enterprises continue to perform complex and nonlinear complementary cooperative activities, and the stronger the interaction, the higher the rate of malicious knowledge sharing and knowledge transmission between nodes. The enterprises continue to find new opportunities, and to develop and promote new innovation results; thus, they enhance the technology innovation performance.

(2) The intelligent manufacturing innovation network (i.e., network relationship, network location, and network density) exerts a significantly positive influence on knowledge absorption capacity. Under the innovation network environment, the open innovation activities of node organizations enable organizations to continuously acquire heterogeneous knowledge, which can enhance the value of the organizations’ existing resources, enhance resource diversity, increase the amount of knowledge possessed by organizations, immensely promote the absorption and transformation of more diverse knowledge by organizations, and enhance the knowledge absorption capacity of organizations [74]. In addition, the innovation network enhances the knowledge conversion rate by enhancing the efficiency pertaining to the node organization of knowledge input and output [73].

(3) Knowledge absorption capacity exerts a partial mediating role in the relationship between the innovation network and technology innovation performance (PF) of intelligent manufacturing enterprises, in which the influence of In-degree (ID) is greater than that of Out-degree (OD). Knowledge absorption capacity refers to the ability of an organization to acquire, integrate, and transform heterogeneous resources continuously and dynamically [26]. Node organizations in the innovation network can utilize their own experience to effectively identify and utilize new heterogeneous knowledge, and the continuous increase of new resources will further promote the exploration, acquisition, and integration of new knowledge, and, thus, enhance the innovation results of the organization. In addition, the ability of an organization to acquire and transform new knowledge is also the source of its own competitive advantage, and knowledge absorption capacity is a scarce competitive resource that can enhance organizational innovation performance [73]. The results also indicate that the network size (SC) exerts no significant influence on the performance of technological innovation, which also indicates that the number of solitary enterprises in the innovation network of the intelligent manufacturing industry are gradually becoming fewer; thus, the cluster intelligent manufacturing industry is developing appropriately. Another crucial observation is as follows: technology turbulence positively regulates the influence that the innovation network exerts on technology innovation performance through the knowledge absorption capacity, which also indicates that the turbulent technological environment promotes exploratory learning and innovation in the current intelligent manufacturing industry.

The practical significance of this study can be explained as follows: although innovation networks exert a promoting effect on enterprise innovation performance and have been widely recognized by scholars, the conclusions obtained from this study provide further guidance for the specific strategies that enterprises should adopt when constructing inter organizational collaborative innovation networks. Based on the aforementioned conclusions, this study offers the following proposals:

(1) The heterogeneity of knowledge in innovation networks promotes cooperation between enterprises and other innovation entities, strengthening the density and intensity of cooperation. As the main source of innovation output, enterprises crucially facilitate innovation activities [34]. Intelligent manufacturing enterprises should strengthen collaboration and interaction with other organizations in the industry; the cluster effect further verifies that isolated enterprises are bound to exhibit a lag in technological innovation development. Enterprises should comprehensively utilize network effects, actively explore novel interaction opportunities, overcome the existing solidified technology resources, introduce novel diversity resources, and exploit the heterogeneity of resources; thus, they can develop novel innovation activities, through which they can enhance the effectiveness of innovation output. Simultaneously, enterprises should actively export technical resources, promote the resource interaction intensity of the innovation network, and promote the overall technological innovation and development of the intelligent manufacturing industry. By acquiring more heterogeneous knowledge, enterprises ultimately enhance the knowledge density of innovation networks and promote industrial upgrading.

(2) Intelligent manufacturing enterprises should consider the enhancement of knowledge absorption capacity as the core path of the innovation network and technology innovation performance transformation, and the top management should crucially focus on the construction and enhancement of organizational knowledge absorption capacity. Specifically, to maintain competitive advantages, intelligent manufacturing enterprises should build scientific strategic development plans, and the leadership should deeply understand the effect of knowledge absorption capacity on long-term organizational development. In daily work plans, they should strengthen the organizational learning ability, actively perform organizational learning activities, and enhance the ability of members to acquire new knowledge. Actively quoting new technical talents is also a crucial method of promoting innovation diversity.

(3) The government should deeply recognize the cluster innovation effect of the intelligent manufacturing industry. The significant influence of network density on technology innovation performance reveals the positive driving force of cluster development on industrial innovation. By utilizing the timely promulgation of relevant policies to promote the development of an intelligent manufacturing cluster, the innovation activities of intelligent manufacturing enterprises are effectively promoted. While encouraging investment in industrial R&D and innovation, the government should clarify the knowledge requirements and differences in innovation capabilities of innovation entities within the industry, comprehensively leverage the innovation driven advantages occasioned by the dynamic nature of innovation network knowledge, and formulate rational policies and systems to actively perform macroeconomic regulation.

## Contributions

This study presents three theoretical contributions. (1) Currently, research on the impact of innovation networks on technological innovation is mainly limited to a certain characteristic attribute of the network, such as analyzing its impact on organizational innovation from the following perspectives: relationship embedding, network location, and knowledge heterogeneity [18–20]. This study divides the innovation network into three dimensions, namely network relationship, network location, and network structure, which are obtained from the overall innovation network perspective. Moreover, the study comprehensively analyzes the impact of innovation networks on enterprise technological innovation and expands the relevant theoretical research scope. (2) The study utilizes knowledge absorption capacity as a mediator to reveal the mechanism by which innovation networks enhance the performance of enterprise technological innovation through knowledge absorption capacity, and it integrates the knowledge creation theory and social network theory, expanding the theoretical content of innovation research. (3) Some studies indicate that technological turbulence influences innovation activities [58–60]. This study considers it as a moderating variable; thus, it explores the moderating effect of technology turbulence on the influence of innovation network on technology innovation performance through knowledge absorption capacity, and further verifies relevant theories.

## Limitations and future direction

This study exhibits the following shortcomings. First, although it utilizes the most authoritative patent data for empirical analysis, the lag of the patent database also poses certain research obstacles. In addition, some advanced technologies, even confidential technologies, often do not apply for public patents; therefore, patent data exhibits certain limitations. Second, the study represents the innovation performance of a company based on the number of applied invention patents. The innovation performance of a company includes many aspects. Future research can further analyze the deep-seated factors of the company’s technological development from perspectives such as the number of novel products developed by the company and the nature of patents. Furthermore, there are many social factors that affect the development of technological innovation (i.e., human initiative, industrial policy environment, and heterogeneity of industry development paradigms). Therefore, how to systematically and comprehensively position the trajectory and internal development mechanism of industrial technological innovation development is also a potential research direction. Finally, this study analyzed only the basic cooperative relationships among network members; however, different types of cooperative relationships exist. Future research can refine the types of cooperation into categories such as industry university research cooperation, state-owned and non-state-owned enterprise cooperation, and Sino–Foreign enterprise cooperation and further analyze the cooperative relationships between network node organizations; thus, the impact that the nature of cooperative relationships exerts on innovation performance and policies can be analyzed.

## Supporting information

S1 Data (RAR)

## References

[1] Feng S, Bernstein WZ, Hedberg T, Barnard Feeney A. Toward Knowledge Management for Smart Manufacturing. Journal of Computing & Information Science in Engineering.2017;17(3):1–24. 10.1115/1.4037178 PMC5615413 28966561

[2] Zhong RY, Xu X, Klotz E, Newman S. Intelligent manufacturing in the context of industry 4.0: a review. Engineering.2017;3(5):616–630.

[3] Zhou J, Li PG, Zhou YH, Liu M. Toward new-generation intelligent manufacturing. Engineering.2018;4 (1): 11–20.

[4] Meng FS, Zhao G. Research on the Factors Influencing the Development of Traditional Manufacturing to Intelligent Manufacturing. Science & Technology Progress and Policy.2018;35 (1): 66–72.

[5] Gao Q, Zhou H. Opportunities, Challenges, and Suggestions for the Intelligent Manufacturing Industry in Beijing. Modern Manufacturing Engineering.2019; 462 (03): 136–141.

[6] Yang L, Zeng DM, Zou SM, Zhao SC. Science Cooperation Network, Knowledge Diversity, and Enterprise Technology Innovation Performance. Studies in Science of Science.2021;39 (5): 867–875.

[7] Li MX, Su JL, Hu C. The impact of enterprise network location and relationship strength on technology innovation performance in industry university research cooperation. Science & Technology Progress and Policy.2020;37 (14): 118–124.

[8] Qu Y, Shi Y, Guo K, Zheng YC. "Has "Intelligent Manufacturing" Promoted the Productivity of Manufacturing Sector?—Evidence from China’s Listed Firms." Procedia Computer Science.2018;139:299–305.

[9] Day CP. Robotics in Industry—Their Role in Intelligent Manufacturing. Engineering.2018;4(4):440–445.

[10] Wang B. The Future of Manufacturing: A New Perspective. Engineering.2018; 4(005):722–728.

[11] Ma S, Zhang Y, Liu Y, Yang H, Ren S. Data-driven sustainable intelligent manufacturing based on demand response for energy intensive industries. Journal of Cleaner Production.2020;274(20):123155.

[12] Gao G, Zhou DM, Tang H, Hu X. An Intelligent Health diagnosis and Maintenance Decision-making approach in Smart Manufacturing. Reliability Engineering and System Safety.2021;216: 107965.

[13] Yang W, Jian R. Research on Intelligent Manufacturing of 3D Printing/Copying of Polymer. Advanced Industrial and Engineering Polymer Research.2019;2(2):88–90.

[14] Yu YB, Zhang JZ, Cao Y, Kazancoglu Y. Intelligent transformation of the manufacturing industry for Industry 4.0: Seizing financial benefits from supply chain relationship capital through enterprise green management. Technological Forecasting and Social Change.2021;172:120999.

[15] Sherlekar R, Starly B, Cohen PH. Provisioned Data Distribution for Intelligent Manufacturing via Fog Computing. Procedia Manufacturing.2019;34:893–902.

[16] Li CQ, Chen YQ, Shang YL. A review of industrial big data for decision making in intelligent manufacturing. Engineering Science and Technology, An International Journal.2021 May;29:101021.

[17] Qi QS, Xu ZY, Rani P. Big data analytics challenges to implementing the intelligent Industrial Internet of Things (IOT) systems in sustainable manufacturing operations. Technological Forecasting & Social Change.2023;190:122401.

[18] Li K, Zhang LM, Fu H, Liu BH, The effect of intelligent manufacturing on remanufacturing decisions. Computers & Industrial Engineering.2023;178: 109114.

[19] Mezgebe TT, Gebreslassie MG, Sibhatoc H, Bahta ST. Intelligent manufacturing eco-system: A post COVID-19 recovery and growth opportunity for manufacturing industry in Sub-Saharan countries. Scientific African.2023;19: e01547. doi: 10.1016/j.sciaf.2023.e01547 36643766 PMC9826537

[20] Zhou Y, Zang JY, Miao ZZ, Minshall T. Upgrading Pathways of Intelligent Manufacturing in China: Transitioning across Technological Paradigms. Engineering.2019;5(4):11.

[21] Xu Z, Dang Y, Munro P. Knowledge-driven intelligent quality problem-solving system in the automotive industry. Advanced Engineering Informatics.2018;38:441–457.

[22] Wang JP. The impact of heterogeneity and stability of network relationship among Chinese manufacturing enterprises on exploratory innovation: the moderating effect of knowledge redundancy. Science Research Management.2020; 41 (11): 92–101.

[23] Markard J, Hekkert M, Jacobsson S. The technological innovation systems framework: response to six criticisms. Environmental Innovation and Societal Transitions.2015;9(16): 76–86.

[24] Wang JP, Yan BT. The impact mechanism of absorptive capacity on organizational efficiency in knowledge intensive organizations Information Science.2020;38 (10): 43–50.

[25] Zack M. Developing a knowledge strategy. California Management Review.1999;41(3): 125–143.

[26] Zahra SA, George G. Absorptive Capacity: A Review, Reconceptualization and Extension. Academy of Management Review.2002;27(2): 185–203.

[27] Xu EM, Zhang H. Research on the Relationship between Enterprise Knowledge Absorption Ability and Performance. Chinese Journal of Management. 2008;5 (6): 841–848.

[28] Moon H, Benedetto AD, Kim SK. The effect of network tie position on a firm’s innovation performance. Journal of Business Research.2022;144:821–829.

[29] Gong YX. The Impact of Network Density and Knowledge Absorption Capacity on Enterprise Innovation Performance. National Circulation Economy.2020;2:82–83.

[30] Xie XM, Zuo LL. The characteristics of enterprises’ collaborative innovation network and innovation performance: a study on the Mesomeric effect based on knowledge absorption capacity. Nankai Management Review. 2013;16(3):47–56.

[31] Song Y, Wang J. Impact of Network Characteristics and Knowledge Attribute on Enterprise Innovation Performance. Management science.2020;33 (3): 63–77.

[32] Ye CS, Chen CM. Collaboration between Industry, University, and Research, Knowledge Absorption Ability, and Enterprise Innovation Performance. Science and Technology Management Research.2022;42 (3):184–194.

[33] Wang HH, Wang MY, Sun YJ. Research on the influencing factors of cross regional collaborative innovation performance between industry and academia from the perspective of social networks. Science and Technology Management Research. 2019;39(3): 26–33.

[34] Li YR and Ma YH. The Influence of Network Characteristics of Regional Innovation Network on Knowledge Innovation Performance. Prediction.2021; 40 (5): 83–89.

[35] Dhanaraj C, Parkhe A. Orchestrating innovation networks. Academy of Management Review.2006;31(3): 659–669.

[36] Sun Y, Zou B, Guo JY, Guo F. Enterprise Innovation in a Technological Turbulent Environment: A Dual Perspective Based on Organizational Redundancy and Learning. Journal of Systems Management.2020;29 (1):41–48.

[37] Bao Y, Chen X, Zhou K Z. External learning, marketdynamics, and radical innovation: Evidence from China’s high-tech firms.Journal of Business Research.2012;65 (8): 1226–1233.

[38] Freeman C. Network of Innovators: A Synthesis of Research Issues. Research Policy.1991;20(5): 499–514.

[39] Ferrary M, Granovetter M. The role of Venture Capital Firms in Silicon Valley’s Complex Innovation Network. Economy and Society.2009;38(2): 326–359.

[40] Liu LJ, Xiang LL. The Evolution Law of Innovation Network Research and Visual Analysis of Hot Fields. Research and Development Management.2019;31 (3): 145–158.

[41] Leite E. Innovation networks for social impact: An empirical study on multi-actor collaboration in projects for smart cities.Journal of Business Research.2022;139:325–337.

[42] Mannak RS, Markus A, Meeus MTH, Raab J, Smit AC. Network dynamics and its impact on innovation outcomes: R&D consortia in the Dutch water sector. Social Networks.2023;74:62–70.

[43] Wu ZC. Network Structure, Innovation Infrastructure, and Regional Innovation Performance: An Analysis Based on the Network DEA Multiplication Model. Journal of Beijing Jiaotong University(Social Sciences Edition).2021;20 (2): 79–89.

[44] Xiong ZD, Wei W, Gu XQ. Network Location, Cross border Search, and Service Innovation Performance of Manufacturing Enterprises. Studies in Science of Science.2020;38 (7): 1304–1316.

[45] Rojas M, Solis E, Zhu JJ. Innovation and network multiplexity: R&D and the concurrent effects of two collaboration networks in an emerging economy. Research Policy.2018;47(6):1111–1124.

[46] Granovetter M. Economic action and social structure: The problem of embeddedness. American Journal of Sociology.1985;91(3): 481–510.

[47] Wang AQ, Xiong SX. Empirical Analysis of the Impact of Enterprise Network relationship on Technology innovation performance. Statistics & Decision.2020; 36 (05): 186–190.

[48] Wang YW, Urban F, Zhou Y, Chen LY. Comparing the Technology Trajectories of Solar PV and Solar Water Heaters in China: Using a Patent Lens. Sustainability.2018;10(11):4166.

[49] Pei YL. Research on the Impact of Industrial and Academic Science Knowledge Transfer on Enterprise Technology Innovation Performance [Ph.D. Thesis]. Xi’an: Xi’an Jiaotong University;2017.

[50] Wan L, Veliyath R, Tan J. Network Characteristics and Firm Performance: An Examination of the Relationships in the Context of a Cluster. Journal of Small Business Management.2012; 51(1):1–22.

[51] Tsai W. Knowledge transfer in intraorganizational networks: effects of network position and absorptive capacity on business unit innovation and performance. Academy of Management Journal.2001;44(5): 996–1004.

[52] Lv YB, Zhu YQ, Bao LL. Introverted Open Innovation and Break through Innovation Performance-The Regulating Effect of Network Location. Scientific Management. 2020; 33 (5):86–100.

[53] Dang XH, Wei L, Yan H. Research on the Impact of Organizational Inertia in Technological Innovation Networks on Dual Innovation. Studies in Science of Science.2016;34 (009): 1432–1440.

[54] Tian ZZ, Wang XH, Sun JY. Innovation Network Structure, Knowledge Transfer and Enterprise Cooperative Innovation Performance. Soft science.2020;34 (11): 77–83.

[55] Zhan K, Shao YF, Tang XM. Research on the impact of network structure of alliance combinations on enterprise innovation capability. R&D Management.2018;30 (6): 47–58.

[56] Lu RY, Zhou YY, Ding YW, Zhou DM, Feng X. Enterprise Innovation Networks: Tracing, Evolution, and Research Prospects. Management World.2021;1:217–233.

[57] Xu L, Li J, Zeng D. How Does Knowledge Network Affect a Firm’s Explorative Innovation? The Contingent Role of R&D Collaborations. Technology Analysis & Strategic Management.2017;29 (9):973–987.

[58] Tsai KH. Collaborative Networks and Product Innovation Performance: Toward a Contingency Perspective. Research Policy.2009;38(5):765–778.

[59] Xu Q. Research on the Mechanism of the Impact of Innovation Openness and Knowledge Absorption Ability on Enterprise Inn-ovation Performance. Forecasting. 2020;39 (5): 9–15.

[60] Cohen W, Levinthal D. Absorptive capacity: a new perspective on learning and innovation. Administrative Science Quarterly.1990;35(1): 128–152.

[61] Sun XM, Cui WT, Wang L. Comprehensive Study on the Relationship between Structural Holes and Enterprise Innovation Performance. Science of Science and Management of S&T. 2014;35 (11): 142–152.

[62] Li Q, Zhang ZH. Structural Hole, Relationship Quality, Knowledge Absorption Ability, and Enterprise Innovation. Journal of Shandong University of Finance and Economics. 2020; 32 (4): 79–88.

[63] Li P, Xue J, Zhou HC. Empirical study on the impact of Knowledge acquisition ability of scientific researchers on their innovation ability. Technology Economics.2015;11 (8): 16–21.

[64] Jaworski BJ, Kohli AK. Market orientation: Antecedents and consequences. The Journal of Marketing.1993;57: 53–70.

[65] Chen LY, Zhou SF, Zou SM, Zeng DM. The impact of technological diversification on corporate performance. Forum on Science and Technology in China. 2016; (3): 88–92.

[66] Cassiman B, Veugelers R. In Search of Complementarity in Inn-ovation Strategy: Internal R& D and External Knowledge Acquisition. Management Science.2006;52:68–82.

[67] Knowledge Thornhill S., innovation and firm performance in high- and low-technology regimes. Journal of Business Venturing. 2006; 21(5):687–703.

[68] Xue J, Zhang ZG. Research on the Relationship between Dyna-mic Learning Ability and Innovation Performance of Enterprises in Technological and Market Environment Turbulence. Science & Technology Progress and Policy. 2015;32 (1): 98–104.

[69] Garud R, Nayyar PR. Transformative capacity: continual struct-u-ring by intertemporal technology transfer. Strategic Management Journal.1994;15:365–385.

[70] Droge C, Calantone R, Harmancioglu N. New Product Success: Is It Really Controllable by Managers in Highly Turbulent Environments? Journal of Product Innovation Management. 2010;25(3):272–286.

[71] Zhang HJ, Shen Y, Zhao XY, Li N. External collaboration heterogeneity internal knowledge network and firm innovation performance. Science Research. 2022; 40 (4): 704–712.

[72] Su Y, Li GP. Green technology innovation ability, product differentiation and enterprise competition: Based on the analysis of listed companies in energy conservation and environmental protection industry. Chinese Journal of Management Science.2021;29 (4): 46–56.

[73] Nonaka, Konno N. The Concept of Ba: Building a Foundation for Knowledge Creation. California Management Review.1998;(4): 40–62.

[74] Zahra SA. Organizational learning and entrepreneurship in family firms: Exploring the moderating effect of ownership and cohesion.Small Business Economics.2012;38(1): 51–65.

